# mSphere of Influence: How I Learned to Love Bacteria and their Tangled Evolutionary Tree

**DOI:** 10.1128/mSphere.00780-21

**Published:** 2021-09-29

**Authors:** Tera C. Levin

**Affiliations:** a Department of Biological Sciences, University of Pittsburgh, Pittsburgh, Pennsylvania, USA

**Keywords:** bacteria, evolution, homologous recombination, microbiology, population genetics

## Abstract

Tera Levin works in the fields of evolution, microbiology, and genetics, studying how adaptation shapes the molecular interactions between eukaryotic hosts and bacterial pathogens. In this mSphere of Influence article, she reflects on how the paper “Population genomics of early events in the ecological differentiation of bacteria” by Shapiro et al. (B. J. Shapiro, J. Friedman, O. X. Cordero, S. P. Preheim, et al., Science 336:48–51, 2012, https://doi.org/10.1126/science.1218198) changed the way she thinks about bacterial gene and genome evolution.

## COMMENTARY

Bacteria do not follow the normal rules of evolution. Why are they such rule breakers? It’s because the rules themselves were formed originally from Darwin, Wallace, and other scientists who were thinking primarily about animals, while being completely unaware of bacterial genetic shenanigans. As a 4th-year graduate student, I too came from this animal-centric world and thought I understood generally how organisms and genomes evolved. But my mind was blown by Shapiro et al., 2012 (1), when I saw for the first time how many of these evolutionary rules are turned upside down by bacteria. It was evolution at warp speed!

First, the “rules” I thought I knew about evolution: in animals, genetic variants (mostly single nucleotide polymorphisms and indels) slowly accumulate over time via mutation, allowing you to make phylogenetic trees showing how organisms are related. Across 10s or 100s of million years, genes sometimes duplicate and diversify into families and, in rare circumstances, horizontal gene transfer allows for genes to be transferred from one species to another. Some genetic variation within species is adaptive, but much is neutral or mildly deleterious, including transposon insertions, altered tandem repeat lengths, etc., that can be used to differentiate recently diverged populations. When certain variants are favored via positive selection, they spread through the population through sexual reproduction and meiotic recombination. Once organisms from different populations are no longer able to sexually reproduce with each other, gene flow is halted, and we consider those organisms to have become distinct species. Most of these “rules” I’d internalized are not incorrect in animals and many other eukaryotes. But in bacteria, it’s a different world.

Less than 20 years after the first bacterial genome was sequenced, Shapiro et al. examined genomic variation across 20 isolates from of a marine bacterium called Vibrio cyclitrophicus. The strains came from two recently diverged populations: populations found on either small marine particles (S strains) or large particles that are likely zooplankton (L strains). As in many bacterial genomes, *V*. *cyclitrophicus* had a core genome consisting of genes shared among all strains, as well as a flexible, accessory set of genes found in some strains but not others. For most genes in the core genome, there was essentially no difference in the alleles between the S and the L strains. All were jumbled together in the phylogenetic tree, suggesting that the genetic variants were easily shared back and forth between S and L habitats. But for four loci spread across distant chromosomal regions, the story was completely different! These genes had dramatically distinct histories from the rest of the genome, such that L and S strains were phylogenetically entirely separated from each other. The accessory genome changed even faster than these four loci did, with entire DNA regions of several kilobases shared frequently within habitats but not between them.

What did this mean? First, rates of genetic recombination and mixing were super high within Vibrio cyclitrophicus, such that there was no tree that could explain >1% of the genome. Instead, a constant churn shuffled genetic variants within and between S and L populations, and did so differently at nearly every locus. There was, in fact, so much gene swapping by homologous recombination that most genetic novelty came from recombination, not from mutation. This sharply contrasted with my expectation that bacteria would reproduce mostly clonally while picking up a few *de novo* mutations along the way, creating a clear species tree of genomic “descent with modification.” Second and even more exciting, a few select genes were incapable of flowing across that S/L barrier. In actuality, the genes probably did physically move into the other habitat but were unable to take hold there, as natural selection for particular genetic variants meant they were successful only within their home environment. As a result, there was a complete halt to gene flow at those four loci and for many of the accessory genes. It was almost as if the genes speciated before the species did! Third, this pattern was possible only at the four special loci because of strong selection. And so, it was these highly consequential regions of the genome that most differentiated the recently diverged populations, rather than neutral alleles. I had no idea that both selection and recombination could be so powerful to allow for only particular selected alleles at one locus while allowing for near-indiscriminate swapping at the genes right next door, totally mixing and matching the set of genes present within each individual. The genetic history truly occurred on a locus-by-locus basis, such that it didn’t even make sense to talk about the evolution of the genome as a whole. As the authors so clearly stated: “Genes, not genomes, sweep populations.”

Although I didn’t know it at the time, this paper was to have a profound impact on my scientific career. Back in 2012, I was studying how certain bacteria could induce multicellular development in eukaryotic plankton called choanoflagellates, with interesting implications for the evolution of multicellularity in animals. While my research was deep in the field of host-microbe interactions, I really considered bacteria only as an external cue that triggered the Actually Interesting Biology. However, after reading this paper, I was drawn in by the strength and speed of bacterial evolution too. I decided that for my future work it would be important to study not just how hosts evolve to respond to bacteria, but rather how hosts and microbes evolve together. I started reading more widely, trying to integrate the parallel field of bacterial evolution with my own roots in eukaryotic evolution. For my postdoc, I went to the lab of Harmit Malik to study molecular arms races that drive adaptation specifically at the host-microbe interface. Now in my own lab, we pursue exactly these questions of how hosts and bacteria evolve together, shaping mechanisms of immunity and pathogenesis along the way. My mind is still regularly boggled by the genetic contortions bacteria can go through and the speed at which they can evolve. With the ever-increasing ease of genome sequencing and continued advances in the field of bacterial evolution, all of us can now see the genetic and evolutionary tricks that bacteria have been playing on us all along.
